# Causal association between gastroesophageal reflux disease and anemia: Mendelian randomization analysis

**DOI:** 10.1097/MS9.0000000000004842

**Published:** 2026-04-01

**Authors:** Yusujiang Tusuntuoheti, Maimaitiaili Maimaitiming, Xudong Huang, Zheqi Zhou, Jiaxin Wen, Kelimu Abudureyimu, Aikebaier Aili

**Affiliations:** a Xinjiang Medical University, Urumqi, China; b Department of Minimally Invasive Surgery, Hernias and Abdominal Wall Surgery, People’s Hospital of Xinjiang Uygur Autonomous Region, Urumqi, China; c Xinjiang Clinical Research Center for Gastroesophageal Reflux Disease and Bariatric Metabolic Surgery, Urumqi, China; d Research Institute of General and Minimally Invasive Surgery, Xinjiang Uygur Autonomous Region, Urumqi, China

**Keywords:** anemia, gastroesophageal reflux disease, hiatal hernia, iron-deficiency anemia, Mendelian randomization

## Abstract

**Background:**

Previous observational studies have indicated a possible link between gastroesophageal reflux disease (GERD) and anemia, but the causal relationships and specific anemia subtypes involved remain unclear.

**Methods:**

To explore the causal relationship between GERD and anemia risk, we conducted a two-sample Mendelian randomization study using the inverse-variance weighting (IVW) analytical method. After stringent selection, 75 and 76 independent single nucleotide polymorphisms were used as instrumental variables for GERD in analyses of anemia and iron-deficiency anemia (IDA), respectively.

**Results:**

IVW analysis demonstrated that genetically predicted GERD was associated with elevated anemia [odds ratios (OR) = 1.35; 95% confidence interval (CI): 1.17-1.56, *P* < 0.001] or IDA risk (OR = 1.30; 95% CI: 1.18-1.42; *P* < 0.001). The results of the weighted median analysis were also consistent with those of the IVW analysis and provided evidence of causal links (*P* < 0.05). However, the sensitivity analyses revealed no evidence of horizontal pleiotropy or heterogeneity.

**Conclusions:**

Our study provides evidence consistent with a causal relationship between GERD and an elevated risk of developing anemia or IDA. However, because all GWAS datasets used in this study were derived from individuals of European ancestry, the generalizability of our findings to other populations remains uncertain and requires confirmation in diverse ethnic groups.

## Introduction

Gastroesophageal reflux disease (GERD) is a troublesome condition characterized by the reflux of gastric contents into the esophagus^[^1^]^. According to a recent study that analyzed the trend in GERD burden across 204 countries over the past two decades, the overall global prevalence of GERD increased by 77.53% from 441.57 million in 1990 to 783.95 million in 2019^[^2^]^. Typical symptoms of GERD include heartburn and regurgitation^[^3^]^ and can also lead to a variety of extraesophageal manifestations such as asthma, cough, throat clearing, and unexplained chest pain, significantly affecting patients’ health-related quality of life^[^4^]^. Recently, observational studies across different age groups have suggested an association between GERD and anemia. For instance, Lupu *et al*^[^5^]^ reported that 13.4% of the children diagnosed with GERD exhibited anemia and reduced serum iron levels. More compellingly, in a large, prospective, population-based study of US adults, a hospital diagnosis of esophagitis was associated with a nearly three-fold increased rate of subsequent hospitalization for iron-deficiency anemia (IDA)^[^6^]^. Despite this strong and consistent observational evidence, the risk of residual confounding remains, making it difficult to establish true causality.

HIGHLIGHTS

- This study marks the first exploration of a genetic relationship between gastroesophageal reflux disease and various types of anemia using a Mendelian randomization approach.
- This study successfully demonstrated that genetic liability to gastroesophageal reflux disease was associated with an increased risk of anemia and iron-deficiency anemia.
- Our findings support the evidence that anemia may be an additional, under-recognized manifestation caused by gastroesophageal reflux disease.
- Our findings highlight the importance of managing gastroesophageal reflux disease for the prevention and treatment of anemia.

Anemia is characterized by insufficient red blood cells, leading to a reduced oxygen-carrying capacity that fails to meet physiological requirements^[^7^]^. Anemia affects approximately one-third of the global population and is associated with higher rates of morbidity and mortality, reduced work productivity, and impaired neurological development^[^8^]^. The adverse health and developmental effects of anemia result from reduced tissue oxygenation, which affects multiple organ systems. For instance, in IDA, diminished iron availability adversely affects brain development and function even before anemia becomes evident^[^9^]^. Understanding the diverse and complex causes of anemia is essential for developing effective interventions. However, the causality of the association between GERD and anemia remains unknown.

Mendelian randomization (MR) is a powerful analytical tool, particularly when randomized controlled trials are infeasible and observational studies are susceptible to bias from confounding variables or reverse causality^[^10^]^. By employing genetic variants as instrumental variables (IV), MR effectively mitigates these biases since alleles are randomly distributed and not influenced by reverse causation^[^11^]^. Therefore, we used a two-sample MR analysis to explore the potential causal effect of GERD on the risk of anemia. Given the increasing use of artificial intelligence (AI) in research and the need for transparent reporting, this study adheres to the TITAN Guideline for reporting AI use in scholarly manuscripts^[^12^]^.

## Methods

This study was carried out in strict accordance with the guidelines of strengthening the reporting of observational studies in epidemiology using Mendelian randomization (STROBE-MR) statement (STROBE-MR checklist)^[^13^]^.

### Study design and data source

**Figure 1.**
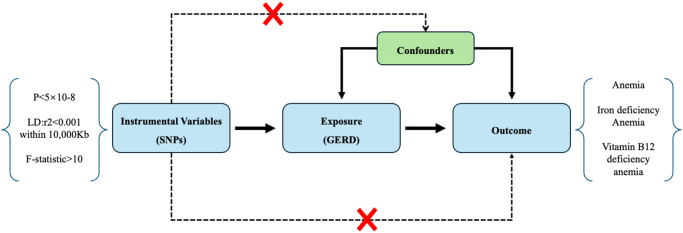
The principles of Mendelian randomization study.

Genetic variants can be used as an IV for a specific exposure if they adhere to the following criteria: they are strongly correlated with the exposure; they are not associated with the outcome through confounding factors; and they influence the outcome only through their effect on the exposure (Fig. 1)^[^10^]^.

Genetic variants data for GERD were derived from the Integrative Epidemiology Unit (IEU) Open Genome-Wide Association Study (GWAS) project (https://gwas.mrcieu.ac.uk/), comprising 129 080 GERD patients and 473 524 controls^[^14^]^. Genetic variants data for anemia and Vitamin B12 deficiency anemia were obtained from a publicly available GWAS dataset in the FinnGen database. Genetic variants data for IDA were obtained from the IEU open GWAS project (Table 1)^[^15^]^. We exclusively sourced all GWAS data from populations of European ancestry. Each GWAS received ethical approval from its respective committee, and the data were freely accessible for use without constraints.

**Table 1:** Data sources and characteristics of GWAS datasets for GERD and anemia phenotypes.

Phenotypes	GWAS ID	Sample Size	Case; Control Sample Sizes	Sex distribution (% female)	Mean age of caes, years	No. of SNPs	Case definition^b^	Sources
GERD	ebi-a-GCST90000514	602,604	129,080; 473,524	NR	NR	2,320,781	Broad GERD phenotype defined from GERD diagnoses and reflux-related symptoms across participating cohorts, as described by Ong *et al* (Gut, 2022)	IEU open GWAS
Anemia	finn-b-D3_ANAEMIA	72,261	12,434; 59,827	57.0%	58.2^a^	16,377,142	FinnGen endpoint D3_ANAEMIA aggregating nutritional, hemolytic, aplastic and other anemias recorded in national health registries	FinnGen
Iron-deficiency anemia	ebi-a-GCST90018872	480,941	12,317; 468,624	NR	NR	480,941	Iron-deficiency anemia defined by clinical diagnosis or ICD codes for iron deficiency anemia (e.g. ICD-10 D50) in the contributing cohorts	IEU open GWAS
Vitamin B12 deficiency anemia	finn-b-D3_ANAEMIA_B12_DEF	212,822	1,707; 211,115	55.8%	58.9^a^	16,380,452	Vitamin B12 deficiency anemia identified by ICD-10 D51 and corresponding ICD-8/9 codes and national reimbursement records	FinnGen

GERD, gastroesophageal reflux disease; NR, not reported.

^a^ Mean age (years) refers to the mean age at first recorded diagnosis/event for cases, as reported in FinnGen summary statistics for the corresponding endpoints (D3_ANAEMIA and D3_ANAEMIA_B12_DEF).

^b^ Case definitions are summarized from the original GWAS publications and FinnGen endpoint definitions.

### Instrumental variables selection criteria

Single nucleotide polymorphisms (SNPs) associated with GERD were selected as IV. To ensure the independence and significance of these SNPs, we applied a linkage disequilibrium (LD) clumping cutoff with an r^2^ threshold of less than 0.001, clumping distance of 10 000 kb, and a genome-wide significance threshold of *P* <5 × 10^−8^. Additionally, instrumental variable strength evaluations (F-statistics) were performed to eliminate weak instruments, and the median F-statistic across all SNPs was 35.16 (31.56, 41.81), indicating strong instruments^[^16^]^. Lastly, to satisfy the independence and exclusion-restriction assumptions, we have removed palindromic SNPs with intermediate allele frequency, and excluded SNPs associated with the outcome at *P* < 5 × 10^−6^.

### Two sample MR analyses

MR analyses were performed using the Two-Sample MR package (version 0.6.8) in R software (version 4.4.0). Potential causality was rigorously evaluated through inverse variance-weighted (IVW), Mendelian randomization Egger (MR-Egger), weighted median, simple mode, and weighted mode. Effect estimates are presented as odds ratios (ORs) with 95% confidence intervals (CIs) per unit increase in the log-odds of genetically predicted GERD.

### Heterogeneity and pleiotropy

Heterogeneity in the analysis was evaluated using Cochran’s Q statistic for both the IVW (fixed effects model) and MR-Egger methods, and quantified using the I^2^ statistics. Heterogeneity was considered absent when the *P*-value from Cochran’s Q was ≥ 0.05. Pleiotropy was evaluated by the MR-Egger intercept method. Genetic variant outliers were identified using MR-PRESSO analysis and leave-one-out analysis was conducted to assess whether any single variant significantly influenced the effect estimates.

## Results

We found 78 SNPs related to genetically predicted anemia and Vitamin B12 deficiency anemia, as well as 80 SNPs related to IDA. No SNPs were excluded based on the MR-PRESSO outlier test or PhenoScanner screening. After harmonizing the exposure and outcome data, three SNPs (rs2358016, rs9517313, and rs957345) were removed from the anemia and Vitamin B12 deficiency anemia-related genetic variants. The remaining 75 SNPs used for the two-sample MR analyses are summarized in (S1 Table). Four SNPs (rs2145318, rs2358016, rs9517313, and rs957345) were removed from the IDA-related genetic variants following the harmonization rules described in the Supplemental Digital Content S1 Methods, available at: http://links.lww.com/MS9/B106; the remaining 76 SNPs used for the two-sample MR analysis are summarized in Supplemental Digital Content Table S1, available at: http://links.lww.com/MS9/B107. This is because they are considered to be palindromic SNPs with intermediate allele frequencies. The median F-statistics of the remaining SNPs was 35.16 (31.56, 41.81), indicating strong instruments.

**Figure 2.**
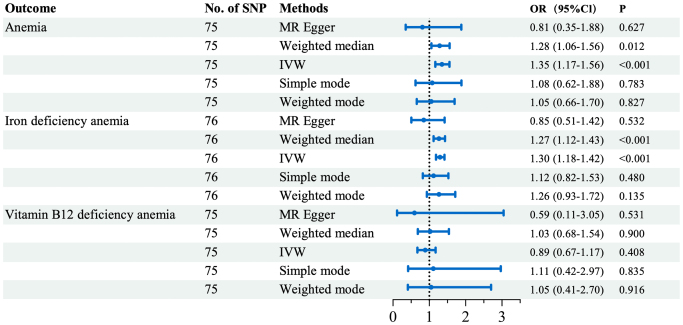
Forest plot of Mendelian randomization estimates for the effect of genetically predicted GERD on each anemia phenotype. MR, Mendelian randomization; SNP, single nucleotide polymorphisms; IVW, inverse-variance weighted; MR-Egger, Mendelian randomization Egger; OR, odds ratio; 95% Cl, 95% confidence interval.

**Figure 3.**
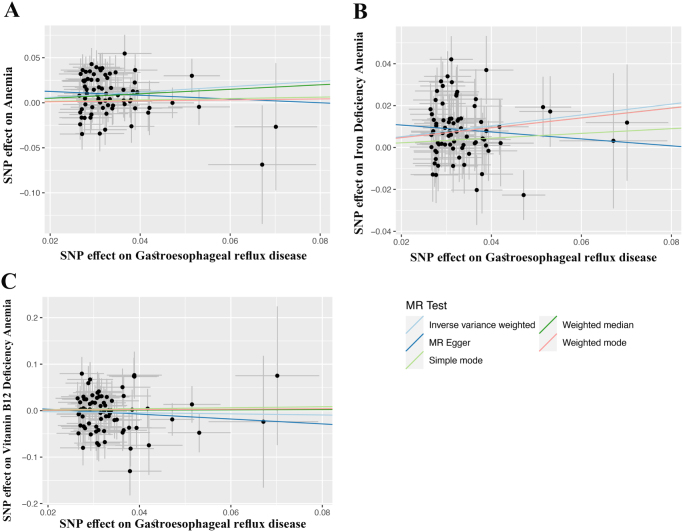
Scatter plots of SNP-specific associations of GERD with (A) anemia, (B) iron-deficiency anemia, and (C) vitamin B12 deficiency anemia. MR, Mendelian randomization; SNP, single nucleotide polymorphisms; MR-Egger, Mendelian randomization Egger.

We assessed the causal relationship between GERD with anemia, IDA and Vitamin B12 deficiency anemia. The IVW analyses suggested that genetic liability to GERD was associated with higher odds of anemia and IDA, whereas no clear effect was observed for Vitamin B12 deficiency anemia (Figures 2 and 3). Per one-unit increase in the genetically predicted log-odds of GERD, the odds of anemia and IDA increased by 35% (OR_IVW_ = 1.35, 95% CI: 1.17-1.56) and 30% (OR_IVW_ = 1.30, 95% CI: 1.18-1.42), respectively. Additional details on statistical power estimation and effect scale interpretation are provided in Supplemental Digital Content S1 Methods, available at: http://links.lww.com/MS9/B106: Power and scale. The weighted median estimates were directionally consistent with the IVW results and remained statistically significant for anemia and IDA (*P* < 0.05), while MR-Egger and mode-based estimators yielded more conservative, non-significant estimates with wider CIs (*P* > 0.05). This pattern – agreement between IVW and weighted median, with less precise MR-Egger estimates – suggests that the findings are not driven by a small number of invalid instruments but should still be interpreted cautiously in light of possible residual pleiotropy and heterogeneity.

However, the analysis did not show any significant intercept (intercept = 0.0168, 0.0140, 0.0135; *P* = 0.2346, 0.1065, 0.6207), providing little evidence of directional horizontal pleiotropy. Cochran’s Q and I^2^ statistics showed low heterogeneity among SNP-specific estimates (Table 2). For anemia, we observed borderline heterogeneity (Q_IVW = 90.88, df = 74, *P* = 0.089; I^2^ ≈ 19%), whereas heterogeneity was very low for IDA (Q_IVW = 79.48, df = 75, *P* = 0.34; I^2^ ≈ 6%) and vitamin B12-deficiency anemia (Q_IVW = 77.96, df = 74, *P* = 0.35; I^2^ ≈ 5%). Such “borderline” heterogeneity for anemia indicates some variability in instrument-specific effects but does not on its own invalidate the causal interpretation, especially when considered alongside the pleiotropy tests and sensitivity analyses.

**Table 2:** Pleiotropy and heterogeneity test.

Phenotypes	Pleiotropy			Heterogeneity							
	MR-Egger			MR-Egger				IVW fixed effects			
	Intercept	SE	*P* value	Q	Q_df	Q_pval	I^2^	Q	Q_df	Q_pval	I^2^
Anemia	0.0168	0.0140	0.235	89.129	73	0.097	18.096	90.883	74	0.089	18.577
Iron-deficiency anemia	0.0140	0.0086	0.107	76.710	74	0.392	3.533	79.478	75	0.340	5.634
Vitamin B12 deficiency anemia	0.0135	0.0273	0.621	77.697	73	0.332	6.045	77.960	74	0.354	5.079

SE, standard error; Q, residual sum of squares; Q_df, the degrees of freedom of residual sum of squares.

No genetic variant outliers were identified using the MR-PRESSO method. In the leave-one-out analysis, no single SNP was identified as having a disproportionately influential effect on causal estimates in a statistically significant manner. Funnel plots and leave-one-out analyses are provided (Supplemental Digital Content Figures: S1–S6, available at: http://links.lww.com/MS9/B105).

## Discussion

This study represents the first MR investigation, providing evidence that GERD elevates the risk of anemia or IDA. Our results indicated that GERD was associated with an increased risk of developing anemia or IDA, with the OR of 1.35 (95% CI: 1.17-1.56, *P* < 0.001) and 1.30 (95% CI: 1.18-1.42, *P* < 0.001), respectively. Although these ORs appear modest, they translate to an absolute risk increase of approximately 7 percentage points for anemia and 2-3 percentage points for IDA, based on typical baseline prevalences. However, no causal relationship was observed between GERD and Vitamin B12 deficiency anemia. GERD is associated with numerous extraesophageal manifestations^[^17^]^. Our findings support the evidence that anemia may be an additional under-recognized common clinical manifestation of patients with GERD.

Our results are consistent with those of several observational reports on the association between GERD and anemia. In an observational study, gastroesophageal reflux was found in 11 of 25 (44%) pediatric patients with refractory IDA^[^18^]^. Blagec *et al*^[^19^]^ reported a case of finger clubbing and IDA associated with severe GERD. However, Shvarts *et al*^[^20^]^ observed that patients with both coronary heart disease and GERD exhibited decreased hemoglobin levels and erythrocyte counts despite the lack of a significant independent effect of GERD. Current research on the relationship between GERD and anemia is characterized by a limited number of observational studies that have yielded inconsistent results. This contributes to an unclear understanding of the causal link between GERD and anemia. Therefore, an MR analysis that specifically addresses this causality is essential.

Hiatal hernia (HH), a common anatomic contributor to GERD, may partly mediate the observed association with anemia through Cameron lesions (CL)^[^21^]^. CL, described as linear ulcerations along herniated gastric mucosa, are a frequent complication of large HH and can lead to chronic blood loss and microcytic anemia^[^22^]^. Although the exact mechanism remains unclear, several variables may contribute to this condition, including reflux esophagitis and diaphragmatic vascular compression^[^23^]^. Addo *et al*^[^24^]^ studied 96 patients with HH. Preoperative identification of CL was observed in 61.5% of patients, and anemia **was present** in 52.5% of these patients. In a systematic review, 58 studies related to CL were identified, and anemia was noted as an initial symptom in 62% of patients^[^25^]^. Consistently, a previous study revealed higher rates of subsequent hospitalization for IDA in patients diagnosed with HH than in those without esophagitis or HH^[^6^]^. Importantly, resolution of anemia and cessation of supplemental oral iron were observed after HH repair^[^24^]^. In our previous study, IDA was observed in 35 (27.8%) of 126 patients with massive HH, and resolution of anemia was achieved in 93.9% of the patients after HH repair surgery^[^26^]^. Haurani *et al*^[^27^]^ investigated 66 anemic patients with paraesophageal hernia, and 85% of those with ulcers or erosions experienced anemia resolution after surgical repair. The resolution of anemia and/or IDA after HH repair underscores the potential relationship between HH and these hematological abnormalities. Furthermore, anemia is associated with increased morbidity and mortality, and a longer hospital stay in patients undergoing paraesophageal hernia repair^[^28^]^. Therefore, physicians should be aware of anemia in the management of HH or GERD.

Beyond mechanical factors such as HH and CL, several additional pathways may link GERD to anemia. First, chronic mucosal inflammation from persistent esophageal acid exposure can alter systemic iron homeostasis. GERD has been associated with elevated circulating pro-inflammatory cytokines^[^29^]^, which can induce hepcidin, a key regulator of iron metabolism. Elevated hepcidin restricts iron absorption and promotes iron sequestration in macrophages, leading to functional iron deficiency and anemia^[^30^]^. Second, dietary adaptations adopted to control reflux symptoms may inadvertently reduce iron intake or bioavailability. For example, low-carbohydrate diets have been shown to improve GERD-related outcomes^[^31^]^, but may increase hepcidin levels and thereby worsen iron restriction^[^32^]^. Therefore, this seemingly beneficial dietary change for GERD could paradoxically contribute to the development of anemia. Third, GERD frequently co-occurs with conditions that themselves predispose to iron deficiency or chronic blood loss, including obesity, prior bariatric surgery, *Helicobacter pylori* infection, long-term use of non-steroidal anti-inflammatory drugs (NSAIDs), and occult gastrointestinal bleeding^[^33–35^]^. These mechanisms likely act in parallel and may also interact with GERD-directed therapies such as long-term acid suppression, further amplifying anemia risk. This highlights the multifactorial nature of anemia in GERD patients and emphasizes the importance of considering these additional pathways, particularly in individuals with severe or refractory symptoms. These results underscore the complex interplay between GERD and anemia, highlighting the need for further investigation into the underlying mechanisms.

Proton pump inhibitors (PPI), as a first-line medical therapy option for GERD, may further exacerbate anemia in patients with GERD, especially those with IDA. Several observational studies have linked long-term PPI use to iron deficiency and reductions in hemoglobin and hematocrit levels^[^36–40^]^. A population-based case-control study found that the adjusted ORs of iron deficiency were 3.60 (95% CI: 3.32-3.91) for individuals with continuous PPI use for ≥1 year and 1.51 (95% CI: 1.44-1.58) for those with intermittent use, compared to individuals who did not receive any PPI prescriptions^[^38^]^. Acid suppression can impair non-heme iron absorption by reducing gastric acidity required for ferric-to-ferrous conversion^[^41^]^. Within an MR framework, PPI use is more plausibly downstream of GERD (a potential mediator) than an upstream confounder; therefore, our MR estimates should be interpreted as the total effect of GERD liability, which may partly operate through treatment patterns. Thus, from a clinical perspective, recognition of these multiple pathways suggests that anemia screening should be considered in patients with long-standing, severe, or treatment-refractory GERD, particularly in those with HH/CL, chronic PPI use, prior bariatric surgery, *Helicobacter pylori* infection, or regular NSAID intake. Conversely, in patients with otherwise unexplained IDA, careful evaluation for GERD and associated HH may help identify a reversible contributor to blood loss or iron malabsorption.

The potential impact of GERD on the development of anemia and IDA has not been adequately explored in epidemiological studies and there is a lack of strong evidence for a causal relationship. Our findings suggest a positive association between GERD and the risk of anemia or IDA. This implies that active management of GERD may help reduce the incidence of anemia or IDA and that addressing GERD could be beneficial for patients with anemia or IDA. In addition, our findings warrant further investigation into the broader implications of GERD on hematological health, including the potential impact on other types of anemia and the long-term consequences of untreated GERD-related anemia. Future studies should also consider the role of dietary and lifestyle factors that might modulate this relationship, providing a more nuanced understanding of how GERD and anemia intersect. However, GERD diagnosis is frequently ignored in patients with anemia; therefore, we recommend active clinical monitoring of GERD in patients diagnosed with anemia.

The use of MR in our study can help to minimize the influence of unobserved confounders and reverse causality. We selected strong IV to ensure F-statistics greater than 10. We established an LD clumping threshold of r^2^ < 0.001 and a distance of <1000 kb from the index variant to ensure data independence. Sensitivity analyses were conducted to enhance the reliability of the results. Additionally, our analyses were well powered, reducing the likelihood that the observed associations were driven by insufficient statistical power. However, this study has several limitations. First, all exposure and outcome GWAS summary statistics were derived from cohorts of European ancestry. Therefore, the estimated causal effects of GERD on anemia and IDA may not be directly generalizable to non-European populations, who differ in genetic architecture, allele frequencies, environmental exposures, dietary patterns, *Helicobacter pylori* prevalence, and healthcare access may modify both GERD risk and anemia susceptibility. Validation of these findings in diverse non-European populations is therefore essential. Second, a further limitation relates to possible sample overlap between the exposure and outcome GWAS. The exposure GWAS for GERD was primarily based on UK Biobank participants, whereas the outcome GWAS for IDA was based on summary statistics from a large cross-population meta-analysis that includes UK Biobank, FinnGen and other cohorts. Thus, some degree of sample overlap between the exposure and outcome datasets is likely, although the exact proportion cannot be determined from the published reports. In the presence of potential sample overlap, two-sample MR estimates can be biased toward the observational association, particularly when instruments are weak. Because all instruments used in our study were strong (F-statistics 30–96) and both GWAS were based on very large biobank samples, any such bias is likely to be modest; however, a small overestimation of the causal effect of GERD on IDA remains possible and the results should therefore be interpreted with appropriate caution. Third, although our results identified a potential causal relationship, the underlying mechanisms require further investigation. Additional well-designed, large-scale studies are necessary to provide more robust evidence and explore the possible mechanisms.

## Conclusion

This is the first MR study to explore the relationship between GERD and anemia. Our findings indicate that genetic liability to GERD is associated with an increased risk of anemia and IDA, consistent with a potential causal effect. These results support the importance of recognizing and managing GERD as a possible contributor to anemia. Given that our analyses were restricted to individuals of European ancestry, replication of these findings in other ancestral groups is essential before extrapolating our conclusions to global populations.

## Data Availability

We utilized GWAS summary statistics from the publicly accessible website “IEU Open GWAS” (https://gwas.mrcieu.ac.uk/), and the data were accessed openly.
